# Treatment Approaches for Refractory *Mycoplasma genitalium* Infection

**DOI:** 10.1093/ofid/ofag031

**Published:** 2026-03-30

**Authors:** Rachel Tenney, Ernesto R Portillo, Danilo Bacic Lima, Julian A Torres Isasiga

**Affiliations:** Albert Einstein College of Medicine, Bronx, NewYork, USA; Department of Internal Medicine, Division of Infectious Disease, Montefiore Medical Center, Bronx, NewYork, USA; Albert Einstein College of Medicine, Bronx, NewYork, USA; Department of Internal Medicine, Division of Infectious Disease, Montefiore Medical Center, Bronx, NewYork, USA; Albert Einstein College of Medicine, Bronx, NewYork, USA; Department of Internal Medicine, Division of Infectious Disease, Montefiore Medical Center, Bronx, NewYork, USA; Albert Einstein College of Medicine, Bronx, NewYork, USA; Department of Internal Medicine, Division of Infectious Disease, Montefiore Medical Center, Bronx, NewYork, USA

**Keywords:** Mycoplasma genitalium, resistance, refractory infections, STI

## Abstract

*Mycoplasma genitalium* is an increasingly recognized sexually transmitted infection (STI) associated with urethritis, cervicitis, and pelvic inflammatory disease. Its management, though, is complicated by rapid expansion of antimicrobial resistance. Because *M. genitalium* lacks a cell wall, treatment options are limited to macrolides, tetracyclines, and fluoroquinolones. Widespread macrolide resistance, along with rising fluoroquinolone and dual-class resistance, has made refractory infection a common and challenging clinical scenario. Current guidelines recommend staged, resistance guided-therapy using a doxycycline lead-in followed by azithromycin for macrolide-susceptible infection or moxifloxacin for macrolide-resistant infection. However, access to macrolide resistance testing remains inconsistent; fluoroquinolone resistance testing is largely unavailable in routine practice, and correlations between resistance markers and clinical failure are incomplete. This review summarizes contemporary standard-of-care treatment strategies and emerging approaches for resistant or refractory *M. genitalium* infection. It also highlights critical gaps in diagnostic capacity, clinical trial data, and the antimicrobial development pipeline for STIs.

*Mycoplasma genitalium* has been increasingly recognized as a bacterial sexually transmitted infection implicated in a growing list of clinical syndromes, including urethritis, cervicitis, and pelvic inflammatory disease, with additional possible associations with pre-term labor and infertility in female patients [1, 2]. However, many questions about *M. genitalium* remain unanswered, for instance, who and when to test, the role of asymptomatic patient screening, causality of syndromes such as proctitis, among others. Concerningly, there has been a rapid expansion of *M. genitalium* antimicrobial resistance making refractory infection a rising clinical problem [3]. The organism lacks a rigid cell wall, making traditional cell wall agents such as penicillins, cephalosporins, and carbapenems ineffective. However, rising macrolide, fluoroquinolone, and tetracycline resistance has eroded first and second-line options from an already limited pool of possible choices, pushing refractory *M. genitalium* from an exception to an expectation [4]. This organism is notoriously difficult to culture and genotypic resistance testing is rarely available in the United States outside research or specialized centers. Unlike gonorrhea, *M. genitalium* has not had a robust surveillance network and a well-developed antibiotic pipeline, and there is no consensus on the clinical management of such refractory infections.

These uncertainties can make clinical encounters with refractory *M. genitalium* uniquely difficult: patients are often frustrated by the lack of clear answers and clinicians are left reconciling what the field currently offers (limited drugs, conflicting guidance, many unresolved questions about clinical significance of the organism in certain populations, limited availability of resistance testing) and what patients who seek sexual health services want (definitive answers, clear guidance, curative treatments).

In this review, we examine the evolution of *M. genitalium* treatment protocols and some emerging approaches for refractory disease, while situating *M. genitalium* as a harbinger of the broader antimicrobial resistance crisis in sexually transmitted infections.

## STANDARD TREATMENT PROTOCOLS

Antibiotics targeting cell-wall biosynthesis such as penicillins and cephalosporins are ineffective against *M. genitalium* as this organism lacks a rigid cell wall. For this reason, antibiotics targeting protein or DNA synthesis, particularly macrolides, tetracyclines, and fluoroquinolones, have been the mainstay of therapy. However, high rates of resistance have resulted in treatment failures, which prompted the adoption of emerging approaches worldwide [1, 5].

The optimal treatment strategy of a *M. genitalium* infection depends on the availability and results of resistance testing. Resistance-guided therapy has shown cure rates exceeding 90% [6, 7] and should be used whenever possible. However, use of a resistance-guided treatment strategy requires access to macrolide resistance testing, which is not currently available at all clinical sites. At present, molecular tests for neither macrolide nor quinolone resistance are commercially available in the US [1]. In the absence of the ability to evaluate for macrolide resistance, empiric 2-staged treatment with doxycycline followed by moxifloxacin is recommended by the CDC [1]. When information regarding macrolide resistance status is available, initial administration of doxycycline is still recommended, with selection of subsequent treatment based on resistance status: infections that are sensitive to macrolides are treated with high-dose azithromycin, while those that are macrolide resistant are managed with moxifloxacin [1, 6, 8]. In all treatment strategies described above, a 2-staged approach is utilized, with doxycycline administered initially with the rationale of reducing bacterial load to facilitate organism clearance with the subsequent treatment [1]. The recommended treatment regimens, based on the availability and results of resistance testing, are summarized in Table 1 below. These regimens are included in the US CDC's most recent STI treatment guidelines, but the emergence of increasing resistance to macrolides and other antibiotic classes, including to quinolones, have prompted both further investigation of additional strategies for the detection of resistant *M. genitalium* infections, and the evaluation of alternative treatment regimens, which are described later in this manuscript.

**Table 1.** Guideline-supported Treatment for *Mycoplasma genitalium* (CDC)

Availability of *M. genitalium* Macrolide Resistance Testing	Result of *M. genitalium* Macrolide Resistance Testing	Recommended Regimen
Available	Macrolide sensitive	Doxycycline 100 mg orally 2 times/day for 7 d, followed by azithromycin 1 g orally initial dose, followed by 500 mg orally once daily for 3 additional d (2.5 g total)
	Macrolide resistant	Doxycycline 100 mg orally 2 times/day for 7 d, followed by moxifloxacin 400 mg orally once daily for 7 d
Unavailable	N/A	Doxycycline 100 mg orally 2 times/day for 7 d, followed by moxifloxacin 400 mg orally once daily for 7 d

Test of cure is not routinely recommended by the U.S. CDC for asymptomatic persons after treatment. Instead, test of cure in the US is recommended for patients with persistent or recurrent symptoms, or when alternative regimens of lower efficacy are used [1]. In contrast, European guidelines advise routine test of cure for all patients, typically performed by Nucleic Acid Amplification Test (NAAT) at least 3 weeks after completion of therapy, given the high prevalence of resistance and limited treatment options [9]. In either framework, guidelines recommend that patients with persistent urethritis, cervicitis, or PID accompanied by detection of *M. genitalium* should receive a fluoroquinolone-based regimen, most commonly doxycycline followed by moxifloxacin [1, 9].

### Treatment in Pregnancy

In pregnancy, treatment options are limited since both doxycycline and moxifloxacin are generally contraindicated. A course of azithromycin (1 g on day 1 followed by 500 mg once daily on days 2, 3, and 4) can be used, though its effectiveness may be compromised in regions with high macrolide resistance. Where available, pristinamycin may also be an option. Management should involve shared decision-making, balancing risks of untreated infection against the risks of treatment [10].

### Partner Management

Studies have shown a high rate of concordant infection among sexual partners [11] and therefore partner notification and evaluation is recommended, though to date there are no prospective studies demonstrating that partner treatment reduces the rate of re-infection. There is insufficient evidence to make a definitive recommendation as to whether asymptomatic sex partners of individuals with *M. genitalium* infection should receive empiric treatment without testing or be treated only if infection is detected through a laboratory test [1, 10, 11]. If partner testing is not possible, empiric treatment with the same regimen used for management of the index partner can be used [1]. Because there are insufficient studies regarding the natural history of such asymptomatic infections, expert approaches vary. The authors of this review recommend disclosure of the many unknowns regarding *M. genitalium* infection and shared decision-making for testing and treatment in such cases.

## DEFINITION AND CAUSES OF REFRACTORY INFECTION

Refractory *M. genitalium* infection is generally defined as the persistence of NAAT positivity and/or clinical symptoms despite appropriate treatment with recommended regimens. In practice, both laboratory and clinical criteria are important: while many patients continue to experience urethritis, cervicitis, or pelvic pain after completing therapy, some may remain asymptomatic, though with persistent NAAT positivity.

Some of the reasons for persistent PCR positivity and ongoing symptoms despite adequate treatment are suboptimal dosage, macrolide resistance, fluoroquinolone resistance, and re-infection through unprotected sex during or after the treatment period.

A 2013 cohort study of young Australian women [12] illustrated the complexity clinicians face in defining “refractory” disease. In practice, without strain typing, sequencing, and drug resistance testing, it can be difficult to distinguish true treatment failure due to resistance, from reinfection after interim clearance, or from persistent infection due to treatment non-adherence.

### Causes of Treatment Failure

Etiologies for failure to treatment have been linked primarily to microbial determinants such as resistance mutations and virulence factors, including enhanced mucosal adhesion and mechanisms that facilitate immune evasion, which may allow *M. genitalium* persistence despite therapy.

Adhesion is mediated by a proteinaceous terminal tip organelle consisting of the MgPa protein and P32 (MG318), which anchor the organism tightly to the epithelial cells of the urethra, cervix, and vagina. The enzymatic activity of glyceraldehyde-3-phosphate dehydrogenase also contributes to adhesion to the vaginal and cervical mucosa, while methionine sulfoxide reductase increases virulence by protecting surface proteins from oxidative damage. Immune evasion is another important mechanism of persistence and is mediated by antigenic variation of the MGPa adhesins, allowing the organism to escape from the host's humoral immune response [13].

Though microbial and host factors are being increasingly understood, rising macrolide, fluoroquinolone, and tetracycline resistance to *M. genitalium* have become the defining challenge in clinical care. Their epidemiology, mechanisms, and perspectives are shared in more detail through the sections below and the other chapters in this supplement.

## MACROLIDE RESISTANCE

### Prevalence

The estimated global prevalence of macrolide resistance exceeds 33% [14], (with resistance rates surpassing 50% in sexual health clinic populations, perhaps due to more frequent exposure to azithromycin) [15]. Prevalence varies widely by region: a 2025 meta-analysis reported the highest estimated resistance rates in the Americas (52%) and Western Pacific region (50%), 38% in Europe, and 0.1% in Africa [14].

### Resistance Mechanisms

Macrolide resistance in *M. genitalium* is driven by single nucleotide polymorphisms (SNPs) in the 23S rRNA gene, most commonly at sites A2058 and A2059 (*E. coli* numbering), which result in disruption of antibiotic binding and a subsequent reduction in efficacy [8, 15–17]. Single nucleotide polymorphisms at the A2058 and A2059 sites develop *de novo*, particularly in the face of selective pressures, such as macrolide exposure. Some evidence has shown that an estimated one in 8 (or approximately 12–18%) of wild type *M. genitalium* infections will develop SNPs yielding macrolide resistance following treatment with a single 1 g dose of azithromycin [5, 17–19]. While *de novo* SNP development is considered the primary driver of macrolide resistance in early phases, over time, the transmission of resistant strains within a community becomes the more significant [20].

## FLUOROQUINOLONE RESISTANCE

### Prevalence

Fluoroquinolone resistance is of growing concern, as rates have increased from an estimated 7% globally pre-2012 to 13%–15% thereafter. The most recent estimate of global fluoroquinolone resistance is 13% [14].

Dual-class resistance to both macrolides and fluoroquinolones, less than 1% before 2014, now reaches an estimated 6.5% globally, with prevalence in some regions surpassing 20%. For example, dual resistance in the Western Pacific region is estimated to be greater than 24% [14]. Rates of macrolide, fluoroquinolone, and dual-class resistance are higher in populations recruited from STI clinics as compared to those recruited at a site other than an STI clinic [14].

### Mechanisms of Fluoroquinolone Resistance

Fluoroquinolone antibiotics exert bactericidal activity by interference with topoisomerase and DNA gyrase, thereby disrupting DNA replication [17]. Fittingly, the most common mechanisms of fluoroquinolone resistance in *M. genitalium* known at this time are mediated by single SNPs affecting the genes coding for topoisomerase IV and DNA gyrase. Mutations in the *parC* gene—most commonly at positions S83 and D87—reduce fluoroquinolone binding to topoisomerase IV, and mutations in the *gyrA* gene— at positions M95I and D99Y—impair binding to DNA gyrase, thereby limiting the ability of fluoroquinolones to stop the replication of *M. genitalium* DNA [15, 17].

Mutations in the *parC* gene are more common than in the *gyrA* gene, though mutations to both can co-occur. In fact, in one Australian study, all isolates with *gyrA* mutations also carried *parC* mutations [21]. Distinguishing the independent contributions of mutations to each gene is challenging given their co-occurrence, though there does seem to be a synergistic effect when mutations to both the *parC* and *gyrA* genes are present. For example, having *gyrA* and *parC* mutations is associated with nearly double the risk of moxifloxacin treatment failure compared to strains with *parC* mutations alone [21].

Resistance to one fluoroquinolone does not necessarily confer resistance to all fluoroquinolones, though there does seem to be some association. For example, a retrospective Australian study found that sitafloxacin-based regimens achieved cure in nearly 70% of cases previously unresponsive to moxifloxacin-based therapy, suggesting partial preservation of efficacy with sitafloxacin, even in the likely presence of *parC* and/or *gyrA* gene mutations. Moxifloxacin treatment failure was however associated with a 6.83-fold increased odds of subsequent sitafloxacin failure [22].

Given that fluoroquinolone's mechanism of action involves disruption of DNA replication, the use of fluoroquinolones could facilitate the development of further fluoroquinolone resistance. By interfering with DNA replication, fluoroquinolones increased mutation rate of *M. genitalium.* Inevitably, some of these mutations will affect the *parC* and *gyrA* genes and may confer resistance [23]. This paradigm underscores the propensity of the organism to acquire resistance under selective antibiotic pressure, highlighting the need for prudent fluoroquinolone use in *M. genitalium* infections.

## DETECTION OF MACROLIDE AND FLUOROQUINOLONE RESISTANCE

*Mycoplasma genitalium* is a fastidious and difficult to culture organism, making culture growth and traditional methods of determining antibiotic susceptibility not feasible. As such, the use of NAAT is a more practical method for detecting antibiotic resistance and guiding therapy. At least 3 commercial assays utilizing NAAT technology exist for the combined detection of *M. genitalium* infection and macrolide resistance [24]. Estimates of sensitivity and specificity for detecting macrolide resistance range across studies and sample type, though generally specificity (96–100%) was found to be higher than sensitivity (70–100%) [24–26]. Assays for detection of macrolide resistance are used more *c*ommonly internationally than in the US, as none are currently cleared by the US FDA for use [2]. As such, macrolide resistance testing in the US is limited to clinical settings connected to reference facilities in which laboratory-developed assays are performed [27].

Detection of quinolone resistance still largely relies on Sanger sequencing, which is not feasible in the clinical setting. However, real-time PCR testing mechanisms are in development and show promising results, with a real-time PCR test for *parC* mutations showing a sensitivity of 100% and specificity of 89.7% in one study [28]. Furthermore, the correlation between quinolone resistance markers and treatment failure is less robust than for macrolides. For example, even with *parC* mutations, moxifloxacin remains effective in approximately 40% of cases [15].

## RESISTANCE GUIDED THERAPY

Given the high prevalence of macrolide resistance, multiple guideline bodies recommend resistance-guided therapy for *M. genitalium* infections [9, 29]. This strategy involves same-day NAAT testing for macrolide resistance mutations at presentation, alongside a 7-day empiric course of doxycycline. Definitive therapy is then tailored to the NAAT result: macrolide-susceptible infections receive a macrolide-based regimen, whereas macrolide-resistant infections are treated with moxifloxacin or sitafloxacin [29]. This approach has achieved cure rates exceeding 90% for both susceptible and macrolide resistant strains of *M. genitalium* [6, 7]. A 2024 cost-effectiveness analysis found resistance-guided therapy to be cost-saving and to increase quality-adjusted life-years (QALYs) in women and men who have sex with men [30].

When more widely available, some experts suggest that *parC* testing may have its greatest clinical value in confirming wild-type sequences. Isolates lacking the S83I mutation demonstrate >95% cure rates with moxifloxacin or sitafloxacin, potentially eliminating the need for a test-of-cure [31]. Conversely, detection of mutations associated with increased risk of treatment failure could prompt closer follow-up, including post-treatment testing and consideration of alternative regimens [32].

## EMERGING SALVAGE STRATEGIES FOR RESISTANT OR REFRACTORY *M. GENITALIUM*

The therapeutic landscape for refractory or resistant *M. genitalium* infection is defined more by uncertainty than by consensus and remains one of the most challenging areas in STI management. Without robust global surveillance and clinical data, large randomized clinical trials or universal resistance testing availability, clinicians are left to extrapolate from small case series, *in vitro* susceptibility data, and from a growing, but imperfect body of observational data. In this setting, designing an individualized salvage regimen is less about following a prescriptive guideline and more about assembling a rational, tolerable, patient-centric and accessible combination of drugs, exerting creativity with caution while setting realistic expectations with patients about the limits of evidence.

Against this background of uncertainty, several strategies, different from those currently recommended by CDC guidelines, have emerged: sequential regimens (eg, minocycline lead-in followed by a fluoroquinolone or macrolide), combination regimens (eg, concurrent tetracycline-quinolone or multi-drug combinations), repurposed antibiotics (eg, pristinamycin, spectinomycin, minocycline, tinidazole, omadacycline), and novel agents (eg, gepotidacin). After a review of the literature, we have summarized the treatment strategies for resistant or refractory *M. genitalium* infection, their availability in the US at present, and the evidence behind each in Table 2.

**Table 2.** Regimens for Standard and Salvage Treatment of *M. genitalium* Infection

Treatment Strategy	US Availability and Approval	Dosing Used in Reports	Evidence and Outcomes
Doxycycline monotherapy	Available	Doxycycline 100 mg BID x 7 d	Cure rates of only 30–40% [33] but is hypothesized to increase the treatment success of the subsequent curative antibiotic by reducing the bacterial load
Doxycycline followed by azithromycin	Both available	Doxycycline 100 mg BID x 7 d → azithromycin 1 g orally initial dose, followed by 500 mg once daily for 3 additional days (2.5 g total)	Prospective cohorts in resistance-guided pathways. Greater than 90% NAAT clearance with macrolide-S. Fails if macrolide-R. De novo resistance risk with azithromycin if no doxycycline lead-in.
Doxycycline followed by moxifloxacin	Both available	Doxycycline 100 mg BID x 7 d → moxifloxacin 400 mg orally once daily x 7 d	Prospective cohort in resistance-guided pathway with 89.5% cure rate; *parC* G248T/S83I mutation present in 76.2% of failures versus 7.8% cures; higher failure rate if concurrent *parC* S83I and *gyrA* mutations than *parC* S83I alone[34]
Doxycycline followed by Sitafloxacin	Sitafloxacin not US-approved	Doxycycline 100 mg BID × 7 d → sitafloxacin 100 mg orally twice daily × 7 d	Retrospective evaluation of 95 cases of macrolide-resistant *M. genitalium,* (>50% failed 1 + prior regimen) with 90% cure; 33% cure rate if prior moxifloxacin failure; prior treatment failure associated with sitafloxacin failure (OR 4.31); prior moxifloxacin failure associated with sitafloxacin failure (OR 6.83) [22]. Prospective cohort in resistance-guided pathway: 89.6% cure rate; *parC* G248T/S83I mutation present in 50% of failures versus 16.8% cures; higher failure rate if concurrent *parC* S83I and *gyrA* mutations than *parC* S83I alone [34].
Concurrent doxycycline + Ssitafloxacin	Sitafloxacin not US approved	Doxycycline and sitafloxacin both 100 mg BID × 7 d	12 cases of macrolide-resistant *M. genitalium,* NAAT positive after moxifloxacin-based/pristinamycin treatment. Greater than 90% with NAAT clearance and complete symptom resolution; 12/12 with *parC* SNPs, 5/12 with *gyrA* SNPs [35].
Doxycycline followed by concurrent Sitafloxacin + Doxycycline (sequential combination therapy)	Sitafloxacin not US approved	Doxycycline 100 mg BID × 7 d followed by combination therapy with doxycycline and sitafloxacin both 100 mg BID for a further 7 d	Sequential combination therapy: Retrospective evaluation of 134 cases of macrolide R *M. gen,* (>50% failed 1 + prior regimen) –> 71.3% cure rate; 71% cure rate if prior moxifloxacin failure [22].
Minocycline followed by fluoroquinolone	Sitafloxacin not available in US	Minocycline 100 mg BID × 7 d followed by either moxifloxacin 400 mg once daily or sitafloxacin 100 mg BID for a further 7 d	Prospective study with 35 patients with macrolide-resistant infection—cure in 25/35 (71%) [36]. Successful case reported treatment of proctitis [37]. Retrospective analysis of a 14-day minocycline monotherapy course—67% cure.
Minocycline and metronidazole	Both available	Concurrent minocycline 100 mg BID with metronidazole 400 mg BID × 14 d	80.8% microbiological cure. CNS and GI side effects commonly reported [38].
Pristinamycin	Not US available (AU/EU use)	1 g, 3–4× daily × 10 d	Prospective/retrospective cohorts. 67–71% NAAT cure. Single-center.
Spectinomycin	Not available in US or Australia. Available on special permit in most of EU.	2 g intramuscularly daily × 7 d	Case study of single individual with microbiologic-proven cure [39] Retrospective case series: 4 patients treated with 2 g IM × 7 d +/− concurrent PO doxycycline, 3 of 4 negative by PCR after 7 d of spectinomycin [40]. Avoidance of monotherapy recommended due to rapid selection for resistance. Consider co-administration with doxycycline 100 mg PO BID × 7 d [41].
Tinidazole	Available	Case report: 2 g PO daily × 7 d. Ongoing clinical trial: after doxycycline 100 mg PO BID × 7 d à 2 g PO on day 1, then 500 mg PO BID days 2–10, with TOC at 21 d	Case reports. Clinical + microbiologic cure after multi-drug failure. Ongoing Phase 2 clinical trial (NCT07088419)
Lefamulin (+/− doxycycline)	FDA-approved for CAP in US. Availability varies.	Lefamulin 600 mg BID × 7 d (monotherapy or following 7-day doxycycline); also 14-d regimens studied	Several studies demonstrating *in vitro* activity. Australian compassionate-use cohort (N = 17) and US open-label RCT (RCT) with inconsistent efficacy across regimens [41–45].
Omadacycline	Available in US	Oral load 450 mg day 1–2 à 300 mg daily (varies)	*In vitro* +/− case-level success [42]. No robust series. GI intolerance, expensive. ID consultation/trial setting preferred.
Zoliflodacin	Investigational. Not approved. Ongoing trials for gonorrhea	N/A	*In vitro* study showed zoliflodacin had greater activity against *M. gen* isolates, including fluoroquinolones, azithromycin, and MDR strains, than moxifloxacin; >75% of isolates S if breakpoint set to MIC < 1 mg/L [46]. No demonstrated cross resistance with fluoroquinolones –> potential candidate specifically for quinolone resistant strains [41].
Solithromycin	Investigational. Not approved	N/A	*In vitro* study showed activity against *M. genitalium* isolates, including isolates with high-level macrolide resistance and multidrug-resistant isolates [47].
Gepotidacin	Investigational. Not approved. Late-phase trials for other STIs.	N/A	*In vitro* studies showed gepotidacin active against all 54 *M. genitalium* isolates tested, median MIC 0.125 mg/L; no difference between macrolide-S and macrolide-R isolates, or between fluoroquinolone, dual –R versus S isolates. *In vitro* synergy w/doxycylcine noted [48]. *In vitro* study showing gepotidacin active against all 10 *M. genitalium* isolates tested; (MIC50 of 0.032 ug/ml); no resistant isolates tested [49].

Abbreviations: BID, twice daily; CAP, community acquired pneumonia; GI, gastrointestinal; IM, intramuscular; IV, intravenous; MDR, multidrug resistant; MIC, minimum inhibitory concentration; NAAT, nucleic acid amplification test; N/A, not applicable; PO, by mouth/oral; QD, once daily; R, resistant; S, susceptible; TOC, test of cure.

### Sequential Therapies

While doxycycline alone is insufficient in achieving clinical and microbiologic cure of *M. genitalium* infections, with cure rates of only 30–40% [33], doxycycline reduces the bacterial load of *M. genitalium* and is hypothesized to thereby increase the treatment success of the subsequent curative antibiotic. Additionally, there is some evidence showing lower rates of selection for macrolide resistance when doxycycline is administered prior to azithromycin [7].

Using Minocycline followed by sitafloxacin or moxifloxacin as alternative to the standard sequential therapy regimen has yielded cure rates of 90%. As is the case for doxycycline, it is hypothesized that minocycline functions by decreasing bacterial load [50].

The relative benefits of concurrent versus sequential administration of a tetracycline and a quinolone remain uncertain. An Australian case series reported outcomes for 12 patients with macrolide-resistant *M. genitalium* who received concurrent doxycycline and sitafloxacin after prior failure of sequential doxycycline–moxifloxacin therapy and a pristinamycin-based regimen [35]. After 7 days of concurrent treatment, 11 of 12 patients achieved both clinical and microbiological cure. Whether this success reflects the concurrent administration, or higher efficacy of sitafloxacin compared with moxifloxacin, is unclear. A retrospective Australian study comparing sequential and concurrent sitafloxacin-based regimens found no statistically significant difference in treatment outcomes, though interpretation was limited by small sample size [22].

### Repurposed Antibiotics

#### Minocycline

The role of minocycline in the treatment of *M. genitalium* infection is an area of active investigation. In a 2020 prospective study of 35 patients with macrolide-resistant infection—68% of whom had received prior antibiotic therapy—minocycline achieved microbiological cure in 71% (25/35) of cases [36]. Similar results were reported in a 2023 retrospective analysis, in which a 14-day minocycline course yielded a 67% cure rate among patients with macrolide-resistant strains [51].

A 2025 prospective study evaluated 104 participants receiving minocycline in combination with metronidazole for cases in which fluoroquinolones were contraindicated or had previously failed [38]. Overall microbiological cure was approximately 80%. When stratified by prior doxycycline exposure, cure rates were 77% without doxycycline pretreatment and 90% with pretreatment, although this difference was not statistically significant.

These data derive from a single sexual health clinic in Australia and thus generalizability to other populations and settings remains uncertain.

More extensive combination regimens including minocycline are being explored for the management of multidrug-resistant *M. genitalium*. A small Belgian case series described 5 individuals with urethritis due to isolates resistant to both macrolides and fluoroquinolones who were treated with a 14- to 28-day course of minocycline, metronidazole, methenamine, and pristinamycin. All patients achieved both clinical and microbiological cure. The individual contributions of each agent in the ultimate cure remain unclear, though the authors hypothesized that some agents may have reduced bacterial load, thereby enhancing the activity of other agent [52].

### Pristinamycin

Pristinamycin has demonstrated efficacy in the treatment of *M. genitalium* infection, particularly in cases with combined macrolide and fluoroquinolone resistance with cure rates of approximately 75% [36, 53]. The efficacy is lower than the estimated 89% cure rate of moxifloxacin [53, 54], and therefore its use is considered primarily after moxifloxacin failure or when fluoroquinolones are contraindicated—such as during pregnancy. Access to pristinamycin is limited; however, despite widespread use in Australia and parts of Europe, the drug is not currently available in several regions, including the US and Japan.

### Spectinomycin

Spectinomycin, an aminocyclitol antibiotic structurally related to aminoglycosides, inhibits bacterial protein synthesis by binding to the 30S ribosomal subunit [40, 41]. Its use in *M. genitalium* infection was first reported in 2016 in a case study describing microbiological cure following daily intramuscular (IM) administration of 2 g for 7 days [39]. Subsequent evidence includes a Japanese retrospective study of four male patients treated with the same regimen, three of whom achieved cure [40].

Spectinomycin is considered safe in pregnancy, but its clinical utility is limited by the need for daily—and often painful—IM injections, a low barrier to resistance when used as monotherapy, and restricted availability in several regions, including the US and Australia [41].

### Tinidazole

A case report demonstrated successful treatment of *M. genitalium* urethritis with high-dose Tinidazole for 7 days following unsuccessful treatment with azithromycin, sequential doxycycline then moxifloxacin, and minocycline, suggesting its possible utility in the face of resistant isolates and need for further study [55]. An ongoing Phase 2 clinical trial is evaluating the efficacy tinidazole after standard doxycycline lead-in for men with non-gonococcal urethritis due to *M. genitalium* (NCT07088419).

### Agents With *In Vitro* Activity

Omadacycline [42], zoliflodacin [46], lefamulin [41, 43–45], and gepotidacin [48, 49]—show *in vitro* activity. Apart from a small clinical trial of Lefamulin [43], the evidence supporting the other agents remains limited to *in vitro* evidence.

## CHALLENGES AND RESEARCH GAPS

As has already been mentioned, the limited pipeline of effective antibiotics to target *M. genitalium* limits clinicians' ability to formulate alternative drugs when first line treatments are ineffective. Only a few of those alternatives have been studied in small trials that lack blinding and randomization and do not include larger, diverse groups of the population, which limit generalizability of findings. Geographic variations in resistance patterns also exist, all within the context of a limited ability to perform molecular testing in some countries. Also, surveillance and reporting inconsistencies might be problematic in such locations. With globalization, the spread of resistance is now easier than a few decades earlier, and our capacity to study this phenomenon may lag real-time variations across different regions.

Here we summarize some important challenges and research gaps for the treatment of *M. genitalium* infection.

### Global Variations in Resistance Patterns

Recent data shows there has been a trend toward increased macrolide, fluoroquinolone and dual antibiotic resistance, globally [14]. The global summary prevalence of macrolide resistance mutations from 2018 to 2021 was 33.3%. This has been particularly important for European countries (Nordic countries: from 20.4%, before 2012, to 56.8% in 2018–21 [*P* = .031]; other European countries: 4.6% to 43.2% during the same study periods [*P* < .001]). This has not occurred in other sites, for instance the African region in which no variations were detected, and the most recent prevalence of macrolide resistance was the lowest (0.1%). In addition, a study in the US showed a prevalence of macrolide resistance of 59.1% [56]. In contrast, the global prevalence of fluoroquinolone resistance mutations has not changed significantly over time and was 13.3% in 2018–21. However, prevalence in the European non-Nordic region increased from 1.7% before 2012 to 12.3% in 2018–21 (*P* = .036). No changes in prevalence were seen in the Western Pacific or African regions, and there were insufficient data for analysis for Nordic countries and the Americas. Finally, the global prevalence of dual-class resistance was 6.5%, the highest seen in the Western pacific region (29.0%), and the lowest in the African region (0.0%). These regional variations may reflect differences in local antibiotic prescribing practices—particularly for non-STI infections (eg, quinolone overuse for UTIs or CAP)—as well as the transmission of resistant organisms within sexual networks (eg, among MSM) [57].

### Limited Antibiotic Pipeline and Lack of Randomized Controlled Clinical Trials for Alternatives

Besides macrolides and quinolones (with or without doxycycline), there is a limited number of alternative antibiotics available to treat *M. genitalium* infections, as already mentioned. This has become problematic in an era of rapid development of resistance and increasing global prevalence of infections, not to mention the limitations associated with medication contraindications or potential safety concerns associated with approved antibiotics for certain groups of patients (eg, pregnancy). A limited number of studies have been conducted to date on alternative agents. These are, unfortunately, small and lack randomization and blind comparison. A more detailed description of such studies is described above. A stronger body of evidence, which includes randomized clinical trials, is urgently needed to address this evolving, narrowing pipeline.

### Surveillance and Reporting Inconsistencies

*Mycoplasma genitalium* testing and surveillance is limited, in part because screening is not yet widely recommended, at least in the US. As of now, testing is recommended for symptomatic individuals, especially if there is recurrent or persistent urethritis, cervicitis or PID. *M. genitalium* infection is also not considered a notifiable infection in the US [58], as it is the case for other STIs like gonorrhea, chlamydia, and syphilis. This is consistent with practices adopted in other Western hemisphere countries. A rare exception is Denmark, where *M. genitalium* has been given the same laboratory-based notification status as Chlamydia since 2023. As *M. genitalium* detection is based on NAAT, reporting is scarce or lacking in countries that have limited healthcare resources and in which these technologies are utilized only in selected clinical scenarios. In the absence of a consistent reporting protocol in most countries, reports tend to occur at the individual level and/or from data obtained at sexual health clinics often as part of a research project, especially to monitor antimicrobial resistance. As understanding of *M. genitalium* clinical outcomes and antimicrobial resistance patterns continues to evolve, and as molecular diagnostic technologies become more widely adopted, updated surveillance practices may be implemented both locally and globally in the near future.

## CONCLUSION

Refractory *M. genitalium* infection sits at the intersection of limited drugs, rising resistance, and many unsettled clinical questions. In practice, the best-supported approach remains resistance-guided, staged therapy (doxycycline lead-in, then macrolide or fluoroquinolone depending on susceptibility), yet even in this framework, clinicians must navigate uncertainties.

Moving the field forward will require expanding access to rapid macrolide resistance testing (and, ideally *parC/gyrA* quinolone resistance assays), designing and conducting randomized and comparative trials of both repurposed and novel agents and combinations against resistant strains, and finally a public health effort of surveillance, tracking resistance in real time to develop empiric guidance across regions. Until those gaps close, clinicians should set clear expectations about uncertainty, prioritize shared decision-making, and keep advocating for the tools and data that patients deserve.
